# Supplementary material for: Pyruvate Dehydrogenase Kinase 1 inhibition mediated oxidative phosphorylation enhancement in cartilage promotes osteoarthritis progression
Source: BMC Musculoskelet Disord. 2023 Jul 20;24:597. doi: 10.1186/s12891-023-06585-6 (PMC10357736; doi:10.1186/s12891-023-06585-6)
Supplement: Supplementary file 1 — Additional file 1. [file 12891_2023_6585_MOESM1_ESM.zip › MF.Gsea.1653627316242/GOMF_THREONINE_TYPE_PEPTIDASE_ACTIVITY.html]

Details for gene set GOMF\_THREONINE\_TYPE\_PEPTIDASE\_ACTIVITY[GSEA]

|  || Dataset | 55457\_collapsed\_to\_symbols.cls.cls #OA\_versus\_normal.cls.cls #OA\_versus\_normal\_repos |
| Phenotype | cls.cls#OA\_versus\_normal\_repos |
| Upregulated in class | OA |
| GeneSet | GOMF\_THREONINE\_TYPE\_PEPTIDASE\_ACTIVITY |
| Enrichment Score (ES) | 0.57066184 |
| Normalized Enrichment Score (NES) | 1.7931246 |
| Nominal p-value | 0.009195402 |
| FDR q-value | 0.043121234 |
| FWER p-Value | 0.596 |
Table: GSEA Results Summary

  

Fig 1: Enrichment plot: GOMF\_THREONINE\_TYPE\_PEPTIDASE\_ACTIVITY      
 Profile of the Running ES Score & Positions of GeneSet Members on the Rank Ordered List

  

| SYMBOL | TITLE | RANK IN GENE LIST | RANK METRIC SCORE | RUNNING ES | CORE ENRICHMENT || 1 | PSMB10 | proteasome 20S subunit beta 10 [Source:HGNC Symbol;Acc:HGNC:9538] | 39 | 1.011 | 0.2034 | Yes |
| 2 | PSMB6 | proteasome 20S subunit beta 6 [Source:HGNC Symbol;Acc:HGNC:9543] | 1188 | 0.479 | 0.2120 | Yes |
| 3 | PSMB7 | proteasome 20S subunit beta 7 [Source:HGNC Symbol;Acc:HGNC:9544] | 1368 | 0.448 | 0.2896 | Yes |
| 4 | PSMB9 | proteasome 20S subunit beta 9 [Source:HGNC Symbol;Acc:HGNC:9546] | 1591 | 0.415 | 0.3570 | Yes |
| 5 | PSMB8 | proteasome 20S subunit beta 8 [Source:HGNC Symbol;Acc:HGNC:9545] | 1721 | 0.395 | 0.4276 | Yes |
| 6 | PSMB3 | proteasome 20S subunit beta 3 [Source:HGNC Symbol;Acc:HGNC:9540] | 1813 | 0.382 | 0.4986 | Yes |
| 7 | PSMB2 | proteasome 20S subunit beta 2 [Source:HGNC Symbol;Acc:HGNC:9539] | 2015 | 0.360 | 0.5564 | Yes |
| 8 | BACE1 | beta-secretase 1 [Source:HGNC Symbol;Acc:HGNC:933] | 2597 | 0.291 | 0.5707 | Yes |
| 9 | PSMB1 | proteasome 20S subunit beta 1 [Source:HGNC Symbol;Acc:HGNC:9537] | 3531 | 0.199 | 0.5389 | No |
| 10 | PSMB4 | proteasome 20S subunit beta 4 [Source:HGNC Symbol;Acc:HGNC:9541] | 3860 | 0.169 | 0.5479 | No |
| 11 | ASRGL1 | asparaginase and isoaspartyl peptidase 1 [Source:HGNC Symbol;Acc:HGNC:16448] | 4122 | 0.144 | 0.5570 | No |
| 12 | PSMA4 | proteasome 20S subunit alpha 4 [Source:HGNC Symbol;Acc:HGNC:9533] | 4412 | 0.120 | 0.5591 | No |
| 13 | GGT1 | gamma-glutamyltransferase 1 [Source:HGNC Symbol;Acc:HGNC:4250] | 5177 | 0.058 | 0.5116 | No |
| 14 | PSMB5 | proteasome 20S subunit beta 5 [Source:HGNC Symbol;Acc:HGNC:9542] | 5586 | 0.025 | 0.4851 | No |
| 15 | TASP1 | taspase 1 [Source:HGNC Symbol;Acc:HGNC:15859] | 6048 | -0.008 | 0.4510 | No |
| 16 | PRSS50 | serine protease 50 [Source:HGNC Symbol;Acc:HGNC:17910] | 6780 | -0.061 | 0.4067 | No |
| 17 | GGTLC1 | gamma-glutamyltransferase light chain 1 [Source:HGNC Symbol;Acc:HGNC:16437] | 6916 | -0.073 | 0.4112 | No |
| 18 | GGT5 | gamma-glutamyltransferase 5 [Source:HGNC Symbol;Acc:HGNC:4260] | 9361 | -0.261 | 0.2747 | No |
Table: GSEA details [plain text format]

  

Fig 2: GOMF\_THREONINE\_TYPE\_PEPTIDASE\_ACTIVITY      
 Blue-Pink O' Gram in the Space of the Analyzed GeneSet

  

Fig 3: GOMF\_THREONINE\_TYPE\_PEPTIDASE\_ACTIVITY: Random ES distribution      
 Gene set null distribution of ES for **GOMF\_THREONINE\_TYPE\_PEPTIDASE\_ACTIVITY**
